# The Interactions of CPP–ACP with Saliva

**DOI:** 10.3390/ijms17060915

**Published:** 2016-06-09

**Authors:** Noorjahan Laila Huq, Helen Myroforidis, Keith J. Cross, David P. Stanton, Paul D. Veith, Brent R. Ward, Eric C. Reynolds

**Affiliations:** Oral Health Cooperative Research Centre, Melbourne Dental School, Bio21 Institute, The University of Melbourne, 720 Swanston Street, Melbourne 3010, Australia; laila@unimelb.edu.au (N.L.H.); helen_myroforidis@hotmail.com (H.M.); keith.cross@unimelb.edu.au (K.J.C.); dstanton@unimelb.edu.au (D.P.S.); pdv@unimelb.edu.au (P.D.V.); brentrw@unimelb.edu.au (B.R.W.)

**Keywords:** saliva, pellicle, casein phosphopeptide, enamel

## Abstract

The repair of early dental caries lesions has been demonstrated by the application of the remineralisation technology based on casein phosphopeptide-stabilised amorphous calcium phosphate complexes (CPP–ACP). These complexes consist of an amorphous calcium phosphate mineral phase stabilised and encapsulated by the self-assembly of milk-derived phosphopeptides. During topical application of CPP–ACP complexes in the oral cavity, the CPP encounters the enamel pellicle consisting of salivary proteins and peptides. However the interactions of the CPP with the enamel salivary pellicle are not known. The studies presented here reveal that the predominant peptides of CPP–ACP complexes do interact with specific salivary proteins and peptides of the enamel pellicle, and provide a mechanism by which the CPP–ACP complexes are localised at the tooth surface to promote remineralisation.

## 1. Introduction

Dental caries is one of the most common oral diseases [1,2]. Aciduric and acidogenic bacteria, in plaque, ferment dietary sugars to produce organic acids that demineralise enamel and initiate dental caries [3]. At early stages of dental caries, the hydroxyapatite mineral loss is reversible [2]. Any products that prevent enamel demineralisation and promote remineralisation are described as exhibiting anticaries activity. Dairy products, such as milk and cheese have been reported to exhibit anticariogenic properties [4,5]. The phosphoprotein caseins complexed with calcium and phosphate are the components of dairy products responsible for this anticariogenic activity [6]. The phosphopeptides of casein derived from enzymic hydrolysis are known as casein phosphopeptides (CPP). These bind calcium and phosphate ions through their multiple phosphoseryl residues in an amorphous, bioavailable form [7,8,9]. The binding to mineral ions is pH dependent and increases from pH 4.0 to a maximum at pH 9.0 [10,11]. 

The resulting complexes are known as casein phosphopeptide amorphous calcium phosphate (CPP–ACP) [12]. The complexes incorporating fluoride are known as casein phosphopeptide amorphous calcium fluoride phosphate (CPP–ACFP) [13,14].

CPP–ACP complexes have been reported to inhibit enamel demineralisation and promote remineralisation of early enamel subsurface lesions [15,16,17,18]. They have been commercialised as Recaldent™ and are currently being added to sugar-free chewing gums (Trident Xtra Care (Americas), Recaldent (Japan)) and dental crème (Tooth Mousse and Tooth Mousse Plus (Europe and Australia), MI Paste and MI Paste Plus (Japan and Americas)) [15]. 

In the oral cavity the tooth mineral surface is covered by a layer of salivary proteins, known as the acquired enamel pellicle whose proteome and peptidome have been recently characterised [19,20,21]. During topical application of the CPP–ACP complexes, the CPP first encounter the enamel pellicle. However the interactions of the CPP with the enamel salivary pellicle have not been investigated.

**Figure 1:**
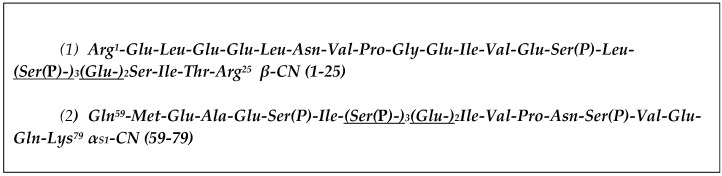
The sequences of the two major casein tryptic phosphopeptides with the calcium phosphate binding-motif underlined are depicted using the three-letter code.

The aim of the study was to investigate the molecular interactions between CPP–ACP complexes, enamel hydroxyapatite, and salivary proteins that would occur during topical application of CPP–ACP in the oral cavity. In this study, a series of experiments revealed that two predominant peptides from CPP–ACP (Figure 1) interact with specific salivary proteins including several that are found within the enamel pellicle. 

## 2. Results

Several *in vitro* approaches were considered to identify the salivary proteins and peptides that bind to both HA and to CPP. In the first strategy, the two predominant peptides of CPP, α_S1_-CN (59–79) and β-CN (1–25), were immobilised separately onto Sepharose 4B for the identification of the salivary peptides and proteins by affinity chromatography. As an alternative strategy, the CPP were immobilised onto HA crystals for identification of bound CPP using SDS-PAGE. A third approach involved adsorbing selected salivary proteins in 96 well plates for the indirect detection by antibodies that recognise the salivary protein-bound CPP. 

### A Diverse Range of Salivary Proteins and Peptides Bind to the Predominant Peptides of CPP–ACP

Affinity chromatography was used to capture the salivary proteins that bound to either α_S1_-CN (59–79) and β-CN (1–25). Whole or parotid saliva was applied to the α_S1_-CN (59–79) or β-CN (1–25) affinity columns following equilibration with 20 mM Tris-HCl pH 8. After removal of the unbound proteins, the bound salivary proteins and peptides were then eluted with a step-gradient of increasing NaCl (0.25–1 M) in 20 mM Tris–HCl pH 8 (Table S1). A blank Sepharose 4B column was used as a control. No salivary proteins or peptides were detected in the 0.25–1 M NaCl eluent of the control column. The affinities of the salivary proteins/peptides bound onto the immobilized CPP columns were qualitatively assessed based on the NaCl concentration required to dissociate the protein. A greater number of proteins from both whole and parotid saliva were identified as interacting with α_S1_-CN (59–79) compared with β-CN (1–25) (Tables S2 and S3). Proteins that bound to both α_S1_-CN (59–79) and β-CN (1–25) included amylase, IgA, histatin, cystatin S, cystatin SN, mucin 7, kallikrein, prolactin inducible protein, Protein S-100-A9, submaxillary gland androgen-related protein 3 homolog B (Tables S2 and S3).

Salivary proteins that eluted from α_S1_-CN (59–79) only with 1 M NaCl include polymeric immunoglobulin receptor (pIgR) (*M*w ~ 65,965; pI 5.14), carbonic anhydrase VI (*M*w ~ 33,569; pI 6.41), Lysozyme C (*M*w ~ 14,700; pI 9.28), and immunoglobulin J (*M*w ~ 15,594; pI 4.59). These tightly bound proteins have masses ranging from 14 to 65 kDa and pI from 4.59 to 9.28. Salivary peptides within the range of 2–5 kDa bound to both α_S1_-CN (59–79) and β-CN (1–25).

**Figure 2:**
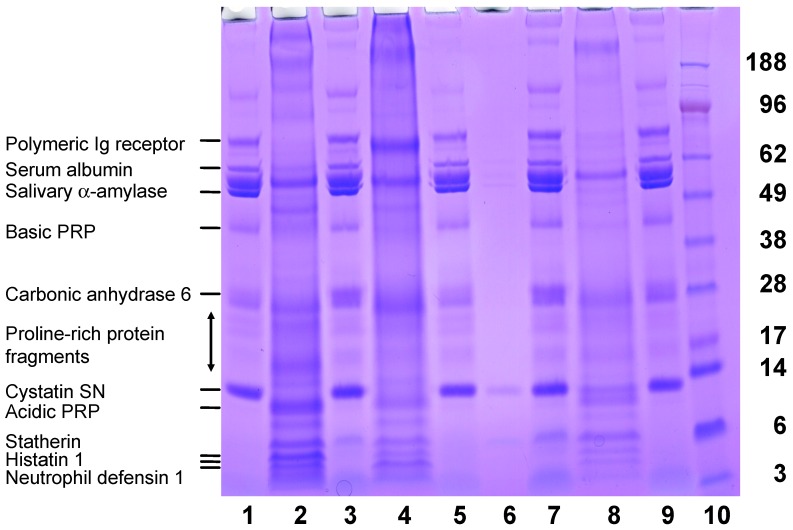
SDS-PAGE profile of salivary proteins derived from whole saliva (WS) bound to casein phosphopeptides (CPP)-coated or uncoated HA. Lane 1: Salivary proteins not bound to β-CN (1–25). Lane 2: Salivary proteins bound to β-CN (1–25). Lane 3: WS. Lane 4: Salivary proteins bound to α_S1_-CN (59–79). Lane 5: Salivary proteins not bound to α_S1_-CN (59–79). Lane 6: WS diluted 1 in 20. Lane 7: WS. Lane 8: Salivary proteins bound to uncoated HA control. Lane 9: Salivary proteins not bound to uncoated HA. Lane 10: Prestained markers.

Another approach was adopted to identify the salivary proteins from whole saliva that adsorbed to HA pre-coated with either α_S1_-CN (59–79) and β-CN (1–25). The bound and unbound salivary proteins were visualised using SDS-PAGE (Figure 2). Observed differences in concentrations of salivary proteins bound to α_S1_-CN (59–79) or β-CN (1–25) immobilised onto HA indicate different affinities. The higher-mass salivary proteins are observed to interact more strongly with α_S1_-CN (59–79)-coated HA as shown in lane 4 (Figure 2). In contrast the low-mass salivary peptides including statherin, histatin 1 and defensin interacted more strongly with the β-CN (1–25)-coated HA as shown in Lane 2 (Figure 2). 

As a control the predominant salivary peptides and proteins derived from whole saliva that bound to HA not coated with the CPP are shown in Lane 8 of Figure 2. These salivary proteins that bound directly to HA were consistent with previous reports on the adsorbed salivary proteins of human acquired enamel pellicle [21,22,23,24,25,26,27,28]. The proteins identified included: bound mucins 5 and 7, amylase, albumin, sIgA, statherin, cystatins, lysozyme, carbonic anhydrase, lactoferrin, lactoperoxidase, histatins, and proline-rich proteins. The peptides identified were: statherin, histatin family, and the proline-rich protein family. The prior adsorption of the CPP (α_S1_-CN (59–79) and β-CN (1–25)) onto HA produced a significant change in the profile of salivary proteins bound (Figure 2 Lane 2 and Lane 4 compared with the control Lane 8). This approach allowed the major proteins/peptides from saliva bound by the CPP to be identified. 

**Figure 3:**
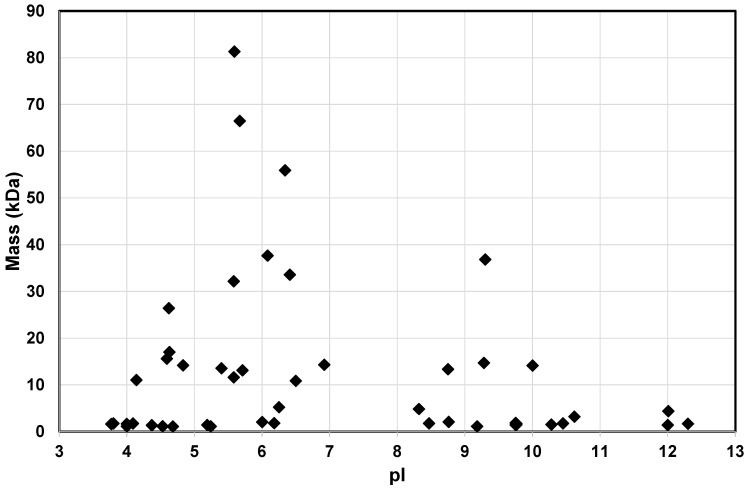
Mass and pI distribution of salivary proteins and peptides of human acquired enamel pellicle that bind to CPP (α_S1_-CN (59–79), β-CN (1–25)).

These two approaches (CPP-sepharose and CPP–HA) identified salivary proteins and peptides that bound to α_S1_-CN (59–79) and β-CN (1–25) that were individually immobilised onto Sepharose 4B matrix or onto HA particles. Analysis of the complete dataset from both approaches confirmed that CPP interacted with selected salivary proteins and peptides known to be found in human acquired enamel pellicle (Table 1 and Table 2) [21,22,23,24,25,26,27,28]. The CPP were found to bind to specific salivary pellicle peptides from the statherin, proline-rich protein and histatin families. Salivary proteins and peptides of pellicle found to bind to CPP exhibited a range of masses and pI values (Figure 3, Table 1 and Table 2). 

**Figure 4:**
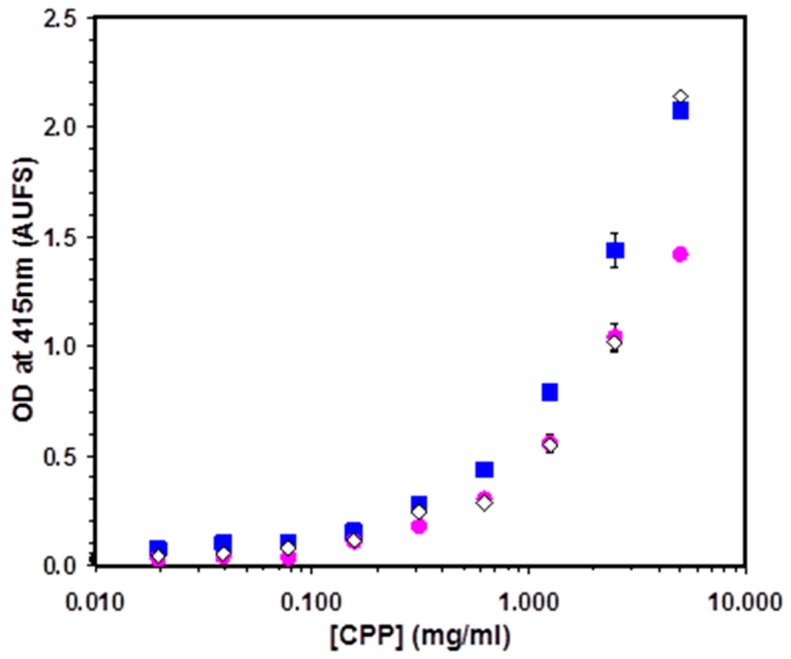
ELISA of α_S1_-CN (59–79) binding to whole saliva (blue squares) and parotid saliva (red circles) and β-CN (1–25) binding to whole saliva (white diamonds).

ELISA was used to observe the binding of α_S1_-CN (59–79) and β-CN (1–25) to adsorbed whole or parotid saliva (Figure 4). Both α_S1_-CN (59–79) and β-CN (1–25) peptides were shown to interact with proteins derived from human saliva.

**Figure 5:**
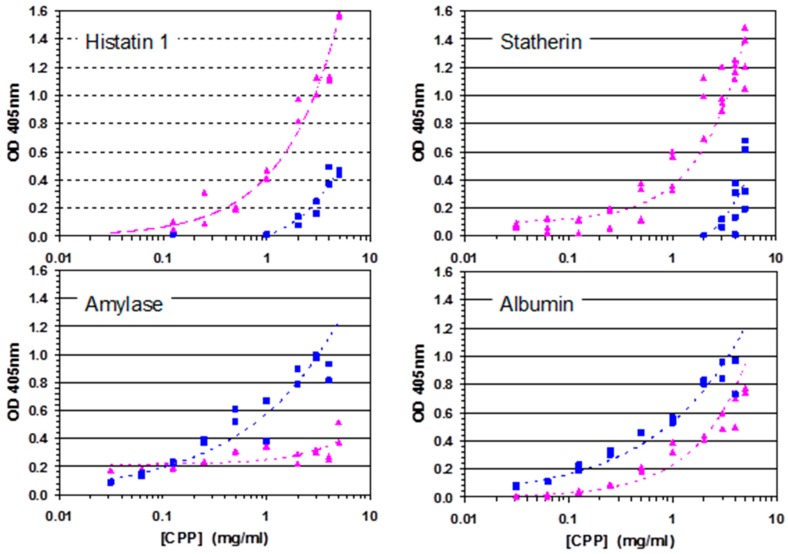
ELISA of α_S1_-CN (59–79) (triangles) and β-CN (1–25) (squares) binding to (**A**) Histatin 1; (**B**) Statherin; (**C**) Amylase; (**D**) Albumin.

To verify the binding of CPP with specific salivary proteins, ELISA was used to observe the dose response with the adsorbed salivary proteins amylase, albumin, and adsorbed peptides Histatin 1, and statherin (Figure 5). The ELISA controls consisted of the anti-casein antibody binding directly to the salivary proteins. Differences in the dose-response curves of the α_S1_-CN (59–79) and β-CN (1–25) were interpreted as differences in affinities for the salivary protein. As observed from the affinity chromatography results, α_S1_-CN (59–79) displayed stronger binding than the peptide β-CN (1–25) to Histatin 1 and Statherin. In contrast to the affinity chromatography results, the β-CN (1–25) displayed stronger binding than the α_S1_-CN (59–79) peptide to both amylase and albumin.

## 3. Discussion

The CPP–ACP complexes commercialised as Recaldent™ are added to dental crèmes for topical application on tooth enamel surfaces to promote enamel remineralisation [15]. It is well established that the CPP and the predominant peptides α_S1_-CN (59–79) and β-CN (1–25) interact with the HA mineral of the enamel both *in vitro* and *in situ* [29]. However, in the oral environment, these dental crèmes are topically applied to the enamel pellicle consisting of salivary proteins and peptides. Hence it is of interest to establish whether the CPP form any protein–protein associations with the pellicle layer to assist in providing mechanistic insight into the remineralisation process. 

Initial ELISA studies confirmed that the two α_S1_-CN (59–79) and β-CN (1–25) peptides recognise proteins in saliva. As several salivary proteins are also phosphorylated, and the milk caseins and salivary statherins and histatins have evolved from a common ancestral gene [30]; it was essential to monitor and subtract the background binding of the antibody to the whole saliva.

This study shows that the two predominant peptides of the CPP have the ability to interact with selected salivary proteins and peptides found as components of the enamel pellicle. The sequences of both α_S1_-CN (59–79) and β-CN (1–25) have hydrophobic and hydrophilic regions. This enables them to bind to a range of proteins through hydrophobic or electrostatic interactions. Nevertheless it is evident that the CPP are not promiscuous binders of salivary proteins, but display selectivity.

The significance of this affinity for specific pellicle proteins is that the remineralisation process of early enamel lesions by CPP–ACP includes protein–protein interactions. These interactions with particular proteins/peptides may facilitate release of the cargo of calcium and phosphate ions carried by the CPP thereby facilitating the remineralisation of the underlying tooth enamel. 

Previous studies have demonstrated the localisation of the CPP within supragingival plaque obtained from humans after treatment with chewing gum or mouth-rinse containing the CPP–ACP [7,8,9]. The CPP were visualised by immunostaining as electron-dense particles associated with the surface of bacteria as well as the intercellular matrix comprised of salivary fluid with salivary proteins and peptides. Furthermore after treatment with CPP–ACP the supragingival plaque showed an increase in both calcium and phosphate levels due to the release of the ions from the complexes as well as the presence of peptides as revealed by immunodetection [7,8,9]. This concomitant retention of mineral ions and peptides derived from the CPP–ACP complexes within plaque on the enamel surface can now be attributed to non-covalent interactions between specific salivary proteins and peptides and the CPP. Hence this study provides the mechanism by which CPP–ACP complexes are localised at the tooth enamel surface to deliver their cargo of calcium and phosphate ions. Fluoride’s anticariogenic mechanism is based on the ion’s ability to drive remineralisation of early carious lesions with fluorapatite [Ca_10_(PO_4_)_6_F_2_; FA] [31]. As remineralisation with FA requires five calcium ions for every fluoride ion the delivery of calcium ions by the CPP–ACP helps explain the reports that CPP–ACP and F have an additive effect in remineralisation of early carious lesions [14,18].

In summary this study has further elucidated the mechanism of anticariogenicity displayed by the CPP–ACP nanocomplexes involving several molecular interactions between organic and inorganic molecules. These interactions include the binding of the CPP to specific salivary proteins and peptides of the enamel pellicle and supragingival plaque [7,8,9]; and release of calcium and phosphate ions from the nanocomplexes to the enamel subsurface lesion [15].

## 4. Experimental Section

### 4.1. Purification of α_S1_-CN (59–79) and β-CN (1–25) Peptides

The α_S1_-CN (59–79) and β-CN (1–25) were purified from a CPP–ACP preparation (Mondelez International, Scoresby, Australia). CPP–ACP (15 mg/mL) in 50 mM EDTA was run on an Agilent 1100 HPLC system using a semi-preparative C18 5 μm column (250 mm × 10 mm VYDAC). Solvent A was 0.1% TFA in deionised water and solvent B was 0.1% TFA in 80% acetonitrile of HPLC grade, and 20% deionised water. The gradient applied for separation was 5% Buffer B for 1 min, 12% at 5.6 min, 36% at 38 min, 100% at 39 min and 100% at 45 min. The gradient was applied over a 45 min interval at a flow rate of 3.5 mL/min with the eluent monitored at 225 nm. Fractions corresponding to the α_S1_-CN (59–79) and β-CN (1–25) peaks were identified on a MALDI-TOF/TOF mass spectrometer (Ultraflex TOF/TOF, Bruker Daltonics, Bremen, Germany) in the positive ion mode. These fractions were pooled, analysed for purity, lyophilised, and stored at −80 °C.

### 4.2. Preparation of Affinity Columns Based on α_S1_-CN (59–79) and β-CN (1–25) 

The α_S1_-CN (59–79) and β-CN (1–25) peptides were separately dissolved in a coupling solution of NaHCO_3_ (0.1 M, pH 8.3) and NaCl (0.5 M), at a concentration of 5 mg/mL. This peptide coupling solution (1 mL) was mixed with Sepharose 4B affinity beads (0.5 g) which had been previously washed with cold HCl (1 mM) for the removal of preservatives. The pH of the solution was adjusted to 8 and coupling of the peptide to beads was achieved by mixing with end-over-end rotation for 2 h at room temperature. Following coupling, the beads were rinsed with NaHCO_3_ (0.1 M, pH 8.3 and NaCl (0.5 M) and blocked for 2 h at room temperature with a solution containing Tris-HCl (0.1 M, pH 8). Following blocking, beads were washed with alternating basic and acidic solutions of Tris–HCl (0.1 M) with NaCl (0.5 M), and Sodium acetate (0.1 M) with NaCl (0.5 M, pH 3), respectively. Coupled beads were packed into empty columns and stored at 4 °C in ethanol (20%).

### 4.3. Adsorption and Elution of Salivary Proteins Bound to α_S1_-CN (59–79) and β-CN (1–25) Columns 

Saliva (whole or parotid) was applied to the α_S1_-CN (59–79) and β-CN (1–25) affinity columns several times to allow for maximum adsorption of salivary proteins to the α_S1_-CN (59–79) and β-CN (1–25) peptides. Un-adsorbed salivary proteins were washed off the columns with Milli-Q H_2_O. Adsorbed salivary proteins were eluted from the columns with increasing concentrations of NaCl (125 mM to 1 M). 

The masses of the salivary proteins and peptides eluted from the α_S1_-CN (59–79) and β-CN (1–25) columns were determined by mass spectrometry [30]. 

### 4.4. Adsorption of CPP to Hydroxyapatite

Purified α_S1_-CN (59–79) and β-CN (1–25) (1000 µg/mL) in 25 mM NaCl and 25 mM imidazole buffer (pH 7) to a total volume of 1 mL were incubated with end-over-end rotation for 4 h at 37 °C with 2 mg of HA (≤200 nm). Following equilibration, samples were centrifuged at 10,000× *g* for 15 min to pellet the peptide bound to HA. The supernatant containing unbound peptide was removed. 

### 4.5. Adsorption of Saliva to CPP-Coated Hydroxyapatite

Whole and parotid saliva was added to HA, coated with either α_S1_-CN (59–79) or β-CN (1–25). Salivary proteins were adsorbed onto hydroxyapatite with some modifications from the method of Jensen and colleagues [32]. Saliva was added to HA in a ratio of 2 mg/mL, mixed and incubated with end-over-end rotation at 37 °C for 2 h. Following the incubation, samples were centrifuged at 14,000× *g* for 15 min at room temperature to pellet the CPP-coated HA and adsorbed salivary proteins. The supernatant was separated from the pellet, which was then washed with 0.1 M NaCl.

The HA was dissolved with 0.2 M EDTA pH 7.5 and incubated for 4 h at 37 °C with end-over-end rotation. After dissolution of the HA the proteins were removed from the mixture by solid phase extraction using C18 SepPak cartridges (Waters, Milford, DE, USA), as described previously. Samples were lyophilised and stored at −80 °C. The protein/peptides of the samples were identified using mass spectrometry [30].

### 4.6. Sample Preparation and SDS-PAGE

Lyophilised samples were resuspended in Milli-Q (Merck Millipore, Bayswater, Australia) H_2_O (20 μL). These samples were added to 4× NuPAGE^®^ LDS Sample Buffer (5 μL) (106 mM Tris HCl, 141 mM Tris base, 2% LDS, 10% Glycerol, 0.51 mM EDTA, 0.22 mM SERVA^®^ Blue G250, 0.175 mM Phenol Red, pH 8.5; Sigma-Aldrich, St Louis, MO, USA). NuPAGE^®^ Reducing Agent 10× (2 μL) (Thermofisher Scientific, Melbourne, Australia) was added to the samples which were made up to a total volume of 27 μL. Samples were heated at 70 °C for 10 min for denaturation of proteins prior to loading on gels. NuPAGE^®^ MES SDS (Thermofisher Scientific, Melbourne, Australia) running buffer was diluted from a 20× stock containing 50 mM MES, 50 mM Tris base, 0.1% SDS, 1 mM EDTA, pH 7.3. Samples and standards were loaded onto 4%–12% NuPAGE^®^ Novex^®^ Bis-Tris Mini Gels (Invitrogen, Melbourne, Australia) and electrophoresis was performed using a Novex Mini X-Cell Surelock™ apparatus (Thermofisher Scientific, Melbourne, Australia) run at 200 V for 35 min.

Gels were stained with 0.1% (*w*/*v*) Coomassie Brilliant Blue R250 in 40% (*v*/*v*) ethanol and 10% (*v*/*v*) acetic acid, for a minimum of three hours in order to visualise the protein bands. Destaining of gels with 10% (*v*/*v*) acetic acid was carried out over 2 days for optimum development of proline-rich proteins, which are observed as pink bands following destaining with acetic acid. Electrophoretic gel bands were subjected to in-gel trypsin digestion, followed by the extraction of the tryptic peptides and subsequent analysis using RP-HPLC coupled to an ESI-TOF/TOF mass spectrometer (Bruker Daltonics, Bremen, Germany) [30].

### 4.7. Collection of Whole and Parotid Saliva

Approval for this study was obtained from the Ethics Committee of The Faculty of Medicine, Dentistry, and Health Sciences of The University of Melbourne (registration ID 000069.1, 2006). Informed consent was obtained from donors prior to saliva collection. Whole unstimulated saliva was collected from healthy adult male and female volunteers. Parotid saliva was collected with the use of a Lashley cup [33]. Following collection, the samples were immediately centrifuged at 14,000× *g* for 15 min at 4 °C for the removal of cell and food debris. The supernatant was collected and 5 µL of Protease Inhibitor Cocktail was added per 1 mL of saliva supernatant to inhibit proteolytic cleavage. The samples were then immediately stored at −70 °C until further use.

### 4.8. Purification of Salivary Proteins

Salivary histatins 1, 3, and 5, as well as statherin, were isolated from human parotid saliva according to the method of Flora [34]. Briefly, parotid saliva was made up with zinc chloride to a final concentration of 500 μM, and the pH was raised to 9.0. The samples were incubated for 20 min at 4 °C. Following incubation, samples were centrifuged at 16,000× *g* for 20 min. The supernatant was removed, and the pellet, which contained the proteins of interest, was washed with 500 μM zinc chloride. Washed pellets were dissolved in Milli-Q water with the addition of 1 M HCl, to a final pH of 2.0 and lyophilised.

Lyophilised samples were dissolved in 0.1% TFA (*v*/*v*) and filtered through a 0.2 μm membrane prior to being subjected to RP-HPLC. Buffer A was 0.1% TFA in Milli-Q water, and Buffer B was 0.1% TFA, 80% acetonitrile and 19.9% Milli-Q. A linear gradient of 0%–55% Buffer B was used over a 74 min interval, with a flow rate of 1 mL/min, and a wavelength of 230 nm was used to monitor the eluate. Collected peaks were lyophilised.

### 4.9. ELISA

The 96-well flat bottomed polyvinyl microtitre plates (Microtiter, Dynatech Laboratories, El Paso, TX, USA) were coated with 50 μL of clarified whole saliva or parotid saliva (10 μg/mL) in Phosphate buffered saline (PBS) and incubated at 4 °C overnight. The excess coating solution was removed and the wells were blocked with 200 μL of 1% (*w*/*v*) BSA in PBS with 0.05% Tween 20 (PBST) for 1 h at RT. The plate was washed four times with PBST. The CPP ((α_S1_-CN (59–79) or β-CN (1–25)) (5 mg/mL) serially diluted in PBS was added to the wells. After incubation for 3 h at RT, the plate was washed four times with PBST and 50 μL of polyclonal rabbit anti-bovine casein antibodies (1:640) in 0.5% (*w*/*v*) BSA was added and incubated overnight at RT. Six washes were performed with PBST. The horse radish peroxidase-conjugated goat anti-rabbit antibody (1:2000) (100 μL) in 0.5% gelatin was added to the wells. After incubation for 3 h at RT, the plate was washed six times with PBST. The substrate solution (100 μL) was added to each well. After 15 min at RT, the optical density (OD) was measured at 415 nm using a Labsystems iEMS MF Ascent microplate reader (Pathtech Pty. Ltd., Melbourne, Australia). The salivary proteins amylase, albumin, and immobilised peptides Histatin 1, and statherin were immobilised at 10 µg/mL concentrations. The background OD values obtained using the anti-casein antibody binding directly to the salivary protein were subtracted from OD values obtained with the addition of CPP.

## 5. Conclusions

CPP–ACP complexes are known to inhibit enamel demineralisation and promote remineralisation of early enamel lesions [15]. During topical application of the dental crème containing the CPP–ACP complexes, the CPP first encounter the enamel pellicle. This study shows that the peptides of the CPP–ACP complexes are able to interact with the salivary proteins and peptides that make up the enamel pellicle. These non-covalent associations between the casein peptides and pellicle proteins and peptides have not previously been recognised as an essential step during the remineralisation process.

## Figures and Tables

**Table 1:** A list of known proteins of human acquired enamel pellicle shown to bind CPP.

Protein	Uniprot No.	*M*w	pI
Salivary acidic proline-rich phosphoprotein 1/2 precursor		11,020	4.14
Immunoglobulin J	P01591	15,595	4.59
Kallikrein-1	P06870	26,406	4.62
Salivary acidic proline-rich phosphoprotein 1/2 precursor	P02810	17,016	4.63
Cystatin-S	P01036	14,189	4.83
Prolactin-inducible protein	P12273	13,523	5.4
Zinc-alpha-2-glycoprotein	P25311	32,145	5.58
Ig kappa chain C region	P01834	11,609	5.58
Polymeric-immunoglobulin receptor	P01833	81,349	5.59
Serum albumin	P02768	66,472	5.67
Protein S100-A8	P05109	13,111	5.71
Ig alpha-1 chain C region	P01876	37,655	6.08
Statherin	P02808	5220	6.25
Salivary alpha-amylase	P04745	55,910	6.34
Carbonic anhydrase 6	P23280	33,570	6.41
Protein S100-A9	P06702	10,835	6.5
Cystatin-SN	P01037	14,316	6.92
Histatin 1	P15515	4848	8.32
Cystatin-C	P01034	13,347	8.75
Lysozyme C	P61626	14,701	9.28
Mucin 7	Q8TAX7	36,809	9.3
Submaxillary gland androgen-regulated protein 3 homolog B	B2R564	14,117	10
Salivary acidic proline-rich phosphoprotein 1/2 precursor		4371	12.01

**Table 2:** A list of known peptides of human acquired enamel pellicle shown to bind CPP.

Peptide/Protein	Sequence	*M*_w_	pI
Lactotransferrin (230–243)	ESTVFEDLSDEAER	1616.8	3.77
Myeloperoxidase (726–741)	DFVNCSTLPALNLASW	1746.871	3.8
Corunlin (373–383)	EQGQTQTQPGS	1151.57	4
Protein S100a14 (13–26)	QEFSDVERAIETLI	1638.78	4
AnnexinA1 (13–26)	FIENEEQEYVQTVK	1749	4.09
Peroxiredoxin-5 (54–67)	APIKVGDAIPAVEV	1370.5	4.37
Protein S100-A8 (84–93)	FWELIGEAAK	1160.1	4.53
Statherin (1–9)	DSSEEKFLR	1098.66	4.68
Histone H2A type 1-A (102–115)	TIAQGGVLPNIQAV	1378.6	5.19
Protein S100-A8 (28–37)	NFHQYSVEGG	1133.64	5.24
Statherin (11–28)	IGRFGYGYGPYQPVPEQP	2024.96	6
Histone H2A type 1-D (89–104)	RNDEELNKLLGKVTIA	1796.89	6.18
Acidic PRP ^#^	SPPGKPQGPPPQGGNQPQ	1766.93	8.47
Acidic PRP ^#^	GPPQQGGHQQGPPPPPPGKPQ	2067	8.76
Con 1 ^#^	PQGPPPQGGSKS	1133.3	9.18
Acidic PRP ^#^	GGRPQGPPQGQSPQ	1388.61	9.75
Acidic PRP ^#^	GPPPQGGRPQGPPQGQSPQ	1855.92	9.75
Acidic PRP ^#^	GRPQGPPQQGGHQQ	1462.5	9.76
Acidic PRP ^#^	GPPQQGGHPPPPQGRPQ	1719.9	9.76
Histatin 3 ^#^	DSHAKRHHGYKR	1487.7	10.28
Histatin 3 ^#^	DSHAKRHHGYKRKF	1762	10.45
Histatin 6	DSHAKRHHGYKRKFHEKHHSHRGYR	3191.64	10.62
Acidic PRP	GPPQQGGHPRPPR	1379.72	12
Con 1	GPPRPPQGGRPSRPPQ	1656.8	12.3

^#^ Numbering is ambiguous due to alternative splicing.

## Supplementary Materials

Supplementary materials can be found at http://www.mdpi.com/1422-0067/17/6/915/s1.
