# Supplementary material for: The Interactions of CPP–ACP with Saliva
Source: Int J Mol Sci. 2016 Jun 9;17(6):915. doi: 10.3390/ijms17060915 (PMC4926448; doi:10.3390/ijms17060915)
Supplement: Supplementary file 1 [file ijms-17-00915-s001.pdf]

Supplementary Materials: The Interactions of CPP-ACP with Saliva

Noorjahan Laila Huq, Helen Myroforidis, Keith J. Cross, David P. Stanton, Paul D. Veith, Brent R. Ward and Eric C. Reynolds

Table S1. Protein amount (%) of whole and parotid saliva eluted from α_{S1}-CN (59–79) and β-CN (1–25) affinity columns.

**Saliva**	**Affinity Column**	**[NaCl] (M)**		
		**0.25**	**0.15**	**1**
Parotid saliva	α_{S1}-CN (59–79)	63.5	19.9	16.6
	β-CN (1–25)	60.6	24.2	15.2
Whole saliva	α_{S1}-CN (59–79)	45.4	30	24.5
	β-CN (1–25)	44.2	30.6	25.2

Table S2. Parotid salivary proteins adsorbed to α_{S1}-CN (59–79) and β-CN (1–25) affinity columns.

Protein name	Accession Number	Molecular Weight (Da)	Peptides Matched	Sequence Coverage %	[NaCl] and Mascot Score			Source
					250 (mM)	500 (mM)	1000 (mM)	
**Serum albumin**	P02768	71,317	9	23	157	0	299	α_{S1}-CN (59–79)
**Salivary α-amylase**	P04745	58,415	14	45	240	0	742	α_{S1}-CN (59–79)
					365	224	0	β-CN (1–25)
**Ig α-1 chain C region**	P01876	38,486	10	40	123	90	422	α_{S1}-CN (59–79)
					171	0	0	β-CN (1–25)
**Ig κ chain C region**	P01834	11,773	4	61	50	0	141	α_{S1}-CN (59–79)
					80	0	0	β-CN (1–25)
**Kallikrein-1**	P06870	29,498	2	5	0	0	109	α_{S1}-CN (59–79)
**Protein S100-A9**	P06702	13,291	2	19	0	0	55	α_{S1}-CN (59–79)
					39	0	0	β-CN (1–25)
**Polymeric-immunoglobulin receptor**	P01833	84,459	8	16	0	0	219	α_{S1}-CN (59–79)
**Submaxillary gland androgen-regulated protein 3 homolog B**	B2R564	5806	2	91	152	109	0	β-CN (1–25)
**Zinc-α-2-glycoprotein**	P25311	34,079	2	17	61	0	128	α_{S1}-CN (59–79)
**Immunoglobulin J chain**	P01591	16,041	2	28	0	0	75	α_{S1}-CN (59–79)
**Lysozyme C**	P61626	16,982	2	18	0	0	11	α_{S1}-CN (59–79)
**Histatin 1 ****	P15515	4848	1	42	71	0	74	α_{S1}-CN (59–79)
					162	361	0	β-CN (1–25)
**Statherin ***	P02808	7304	2	47	0	25	0	β-CN (1–25)
**Ig α-2 chain C region**	P01877	37,283	2	13	0	0	291	α_{S1}-CN (59–79)
**Prolactin-inducible protein**	P12273	16,847	2	15	0	107	0	β-CN (1–25)

All scores, except for Statherin, relate to searches against an in-house saliva protein database, and also identified by Swissprot, with at least two top scoring peptides (not necessarily statistically significant). * Identification through tryptic peptides of the protein, identified previously in the literature [34]; ** Identification achieved through one tryptic peptide, however this corresponds to 42% sequence coverage.

Table S3. Whole salivary proteins adsorbed to α_{S1}-CN (59–79) and β-CN (1–25) affinity columns.

Protein Name	Swissprot Accession Number	Molecular Weight (Da)	Peptides Matched	Sequence Coverage %	[NaCl] and Mascot Score			Source
					250 (mM)	500 (mM)	1000 (mM)	
Serum albumin	P02768	68,425	9	17	137	258	408	α_{S1}-CN (59–79)
Salivary alpha-amylase	P04745	56,501	9	26	112	242	395	α_{S1}-CN (59–79)
					116	0	0	β-CN (1–25)
Cystatin-SN	P01037	14,535	2 (2 unique to Cystatin-SN)	23	0	288	0	α_{S1}-CN (59–79)
					300	0	0	β-CN (1–25)
Cystatin-S	P01036	14,408	8 (2 unique to Cystatin-S)	77	245	0	0	α_{S1}-CN (59–79)
					700	102	0	β-CN (1–25)
Ig alpha-1 chain C region	P01876	38,486	7	28	107	192	264	α_{S1}-CN (59–79)
Mucin 7 *	Q8TAX7	39,171	4	4	44	42	66	α_{S1}-CN (59–79)
					36	0	0	β-CN (1–25)
Kallikrein-1	P06870	26,846	2	10	65	62	86	α_{S1}-CN (59–79)
					67	0	0	β-CN (1–25)
Prolactin-inducible protein	P12273	13,742	5	49	67	193	112	α_{S1}-CN (59–79)
					280	0	0	β-CN (1–25)
Protein S100-A9	P06702	13,291	3	35	0	67	0	α_{S1}-CN (59–79)
					75	0	0	β-CN (1–25)
Protein S100-A8	P05109	10,885	2		0	76	0	α_{S1}-CN (59–79)
Zinc-alpha-2-glycoprotein	P25311	32,353	3	12	45	0	133	α_{S1}-CN (59–79)
Carbonic anhydrase 6	P23280	35,459	2	7	0	0	98	α_{S1}-CN (59–79)
Lysozyme C	P61626	16,982	2	18	0	0	95	α_{S1}-CN (59–79)
Cystatin-C	P01034	16,017	2 (2 unique to Cystatin-C)	22	0	131	0	α_{S1}-CN (59–79)
Immunoglobulin J	P01591	16,041	2	20	0	0	51	α_{S1}-CN (59–79)
Ig κ chain C region	P01834	11,773	4	64	0	72	107	α_{S1}-CN (59–79)

Table S3. *Cont.*

Protein Name	Swissprot Accession Number	Molecular Weight (Da)	Peptides Matched	Sequence Coverage %	[NaCl] and Mascot Score			Source
					250 (mM)	500 (mM)	1000 (mM)	
Statherin *	P02808	7304	2	39	0	35	0	α_{S1}-CN (59–79)
					21	0	0	β-CN (1–25)
Histatin 1 **	P15515	4848	1	42	35	187	75	α_{S1}-CN (59–79)
					67	54	0	β-CN (1–25)
Submaxillary gland androgen-regulated protein 3 homolog B ***	B2R564	5806	6	67	197	58	0	α_{S1}-CN (59–79)
					144	0	0	β-CN (1–25)
Salivary acidic proline-rich phosphoprotein 1/2 precursor ***	P02810	17,016	2	27	94	0	0	α_{S1}-CN (59–79)
Polymeric-immunoglobulin receptor	P01833	84,459	4	12	0	0	131	α_{S1}-CN (59–79)

All scores except Statherin and Mucin 7 relate to searches against an in-house saliva protein database, and also identified by Swissprot, with at least two top scoring peptides (not necessarily statistically significant). * Identification through tryptic peptides of the protein, identified previously in the literature [34]; ** Identification achieved through one tryptic peptide, however this corresponds to 42% sequence coverage; *** Identification with semi-tryptic peptides.
